# A MEM1-like motif directs mesophyll cell-specific expression of the gene encoding the C_4_ carbonic anhydrase in *Flaveria*

**DOI:** 10.1093/jxb/erw475

**Published:** 2016-12-31

**Authors:** Udo Gowik, Stefanie Schulze, Montserrat Saladié, Vivien Rolland, Sandra K Tanz, Peter Westhoff, Martha Ludwig

**Affiliations:** 1 Institute of Plant Molecular and Developmental Biology, Heinrich-Heine-University, Düsseldorf, Germany; 2 School of Chemistry and Biochemistry, University of Western Australia, Crawley, WA, Australia; 3 Australian Research Council Centre of Excellence for Translational Photosynthesis, Plant Science Division, Research School of Biology, Australian National University, Canberra, ACT, Australia; 4 Australian Research Council Centre of Excellence in Plant Energy Biology, University of Western Australia, Crawley, WA, Australia

**Keywords:** C_4_ photosynthesis, carbonic anhydrase, evolution of C_4_ photosynthesis, *Flaveria*, gene expression, MEM1, mesophyll cell expression, translatome

## Abstract

The first two reactions of C_4_ photosynthesis are catalysed by carbonic anhydrase (CA) and phospho*enol*pyruvate carboxylase (PEPC) in the leaf mesophyll (M) cell cytosol. Translatome experiments using a tagged ribosomal protein expressed under the control of M and bundle-sheath (BS) cell-specific promoters showed transcripts encoding CA3 from the C_4_ species *Flaveria bidentis* were highly enriched in polysomes from M cells relative to those of the BS. Localisation experiments employing a CA3-green fluorescent protein fusion protein showed *F. bidentis* CA3 is a cytosolic enzyme. A motif showing high sequence homology to that of the *Flaveria* M expression module 1 (MEM1) element was identified approximately 2 kb upstream of the *F. bidentis* and *F. trinervia ca3* translation start sites. MEM1 is located in the promoter of C_4_*Flaveria ppcA* genes, which encode the C_4_-associated PEPC, and is necessary for M-specific expression. No MEM1-like sequence was found in the 4 kb upstream of the C_3_ species *F. pringlei ca3* translation start site. Promoter–reporter fusion experiments demonstrated the region containing the *ca3* MEM1-like element also directs M-specific expression. These results support the idea that a common regulatory switch drives the expression of the C_4_*Flaveria ca3* and *ppcA1* genes specifically in M cells.

## Teaser

*Flaveria bidentis* carbonic anhydrase 3 catalyses the first step in C_4_ photosynthesis, with its cognate gene containing an element that shares homology and function with the C_4_*Flaveria* MEM1 motif.

## Introduction

The C_4_ photosynthetic pathway is an extraordinary example of convergent evolution with more than 65 independent origins among the angiosperms (Sage *et al.*, 2012; Sage, 2016). The pathway functions as a CO_2_ concentrating mechanism (CCM) by increasing the levels of CO_2_ around Rubisco, thereby enhancing the likelihood of CO_2_, rather than O_2_, landing in the active site of the enzyme. This results in C_4_ plants demonstrating reduced photorespiration, increased photosynthetic rates, and greater photosynthetic water and nitrogen use efficiencies relative to C_3_ plants in hot, dry, high light environments (Ghannoum *et al.*, 2011).

Two major groups of land plants using the C_4_ pathway have been described – one group employs both mesophyll (M) and bundle-sheath (BS) cells to fix atmospheric CO_2_, while species in the other group operate a C_4_ cycle in a single cell type (Edwards and Voznesenskaya, 2011). C_4_ species using M and BS cells exhibit Kranz anatomy (Haberlandt 1896), for which at least 25 different forms have been described (Edwards and Voznesenskaya, 2011), but which generally is recognised as vascular tissue surrounded by BS cells, which in turn are surrounded by M cells. Kranz C_4_ species have been categorised further as one of three subtypes (Gutierrez *et al.*, 1974; Hatch *et al.*, 1975; Hatch, 1987; Kanai and Edwards, 1999), based on the decarboxylase that shows the greatest activity in the BS: NADP-malic enzyme (NADP-ME), NAD-malic enzyme or phospho*enol*pyruvate carboxykinase (PCK). In all these C_4_ subtypes, the M cells constitute the photosynthetic carbon acquisition tissue and contain all of the C_4_ form of phospho*enol*pyruvate carboxylase (PEPC), the primary carboxylase in C_4_ plants. All the Rubisco in a C_4_ leaf is in the BS, and therefore, these cells compose the photosynthetic carbon reduction tissue.

While C_4_ plants may differ in their anatomy, primary decarboxylases, and the species of three- and four-carbon acids transferred between the M and BS, the first two reactions of the C_4_ pathway are invariant, and take place in the M cell cytosol. The reactions involve the conversion of atmospheric CO_2_ to bicarbonate (HCO_3_^−^) by the enzyme carbonic anhydrase (CA), and the subsequent utilisation of HCO_3_^−^ by PEPC to form oxaloacetate through the carboxylation of phospho*enol*pyruvate. Depending on the decarboxylase(s) present, the oxaloacetate is rapidly converted to malate and/or aspartate, which then diffuse into the BS where they are decarboxylated, and the released CO_2_ is re-fixed by Rubisco. The three-carbon organic acids resulting from the decarboxylation reaction diffuse back into the M where they can be used in another round of the C_4_ acid transfer cycle.

*Flaveria* is one of a small number of taxa containing species that are C_3_, others that are C_4_, and still others that are C_3_–C_4_ intermediates (Powell, 1978; Edwards and Ku, 1987). This dicotyledonous group has been at the forefront of research into the evolution of C_4_ photosynthesis, not only because it contains multiple C_3_ and C_4_ species, but also because of numerous C_3_–C_4_ intermediate species that essentially form a continuum, representing the stages along the path to the C_4_ syndrome from an ancestral C_3_ state (McKown *et al.*, 2005; McKown and Dengler, 2007; Sage *et al.*, 2012; Heckmann *et al.*, 2013; Lyu *et al.*, 2015). The PEPC and CA isoforms in the leaves of a number of *Flaveria* species are some of the best characterised C_4_-associated proteins with respect to the molecular mechanisms used during evolution that distinguish their cognate gene expression patterns, inter- and intracellular locations, and biochemistry from the ancestral C_3_ homologues (reviewed in Westhoff and Gowik, 2004; Ludwig, 2011).

In *Flaveria*, the gene family coding for PEPC consists of three classes, *ppcA*, *ppcB* and *ppcC* (Hermans and Westhoff, 1992; Ernst and Westhoff 1997), with the C_4_-associated PEPC encoded by the *ppcA* gene (Hermans and Westhoff, 1992; Westhoff and Gowik, 2004). The proteins encoded by orthologous *ppcA* genes from C_3_ and C_4_*Flaveria* congeners show different kinetic and regulatory properties (Svensson *et al.*, 1997; Bläsing *et al.*, 2000). The expression of the C_4_*Flaveria ppcA* gene in the M cytosol requires the M expression module 1 (MEM1), a 41 bp element located in the 2.2 kb region upstream of the *ppcA* translation start site (Gowik *et al.*, 2004). The element is composed of A and B segments, with a guanine residue in the first position distinguishing C_4_ and C_4_-like *ppcA* MEM1 A segments from the orthologues of C_3_ and C_3_–C_4_ intermediate *Flaveria* species, which contain an adenine in the homologous position (Gowik *et al.*, 2004; Akyildiz *et al.*, 2007). Interestingly, a CACT tetranucleotide in the B segment is found in *Flaveria* C_4_, C_4_-like and C_3_–C_4_ intermediate *ppcA* MEM1 elements, but is absent in the upstream region of C_3_*Flaveria ppcA* genes (Gowik *et al.*, 2004; Akyildiz *et al.*, 2007). The MEM1 acts as an enhancer element, conferring M cell-specific reporter gene expression, and in combination with a proximal promoter region (PR) leads to high M expression (Gowik *et al.*, 2004). It also represses gene activity, inhibiting *ppcA* expression in the BS cells and vascular bundles of the leaf (Akyildiz *et al.*, 2007).

Three cDNAs encoding distinct CA isoforms, CA1, CA2, and CA3, have been isolated from the leaves of the C_3_*F. pringlei* and C_4_*F. bidentis* (Tetu *et al.*, 2007; Tanz *et al.*, 2009). *F. bidentis* plants genetically transformed with an antisense construct recognising CA3 mRNA showed reduced levels of total leaf CA activity (von Caemmerer *et al.*, 2004), with transformants containing less than 10% of wild type activity exhibiting a compromised CCM, and a growth requirement for high CO_2_. Transcripts encoding CA3 in *F. bidentis* are at least 50 times more abundant than those coding for CA1 or CA2 (Tetu *et al.*, 2007), and are an order of magnitude greater on a leaf total RNA basis than the transcripts coding for any of the CA isoforms in *F. pringlei* (Ludwig, 2011). Although these transgenic and quantitative analyses suggested CA3 is the C_4_-associated CA in *Flaveria*, and preferential expression is expected in the M to ensure high concentrations of HCO_3_^−^ for PEPC function (Gutierrez *et al.*, 1974; Ku and Edwards, 1975; Burnell and Hatch, 1988), the studies did not resolve whether the high level of *ca3* expression in *F. bidentis* was in fact in a specific leaf cell type. At the protein level, studies using radiolabelled CA precursor proteins indicated that while *F. pringlei* CA3 was imported into isolated pea chloroplasts (Tanz *et al.*, 2009), where CA is required for lipid biosynthesis and stress responses (DiMario *et al.*, 2016), CA3 from *F. bidentis* was not, and was presumed to be a cytosolic form of the enzyme (Tetu *et al.*, 2007), again a result in keeping with the earlier work indicating a cytosolic location of C_4_-associated CA isoforms (Gutierrez *et al.*, 1974; Ku and Edwards, 1975; Burnell and Hatch, 1988). Support for a cytosolic location of CA3 also came from sequence analyses that showed relative to the N-terminus of CA3 from *F. pringlei*, the *F. bidentis* isoform lacks 72 amino acids, which have characteristics of a chloroplast targeting sequence (Tetu *et al.*, 2007; Tanz *et al.*, 2009). Immunocytochemistry using an anti-CA antiserum also supported a location in M cytosol in *F. bidentis*; however, the antiserum was not specific to CA3 (Tetu *et al.*, 2007). CA2, which shows similar transcript abundance in leaves, roots and flowers, and is therefore unlikely to be associated with C_4_ photosynthesis, also localises to the cytosol in *F. bidentis* (Tetu *et al.*, 2007) and may have been immunolabelled. Nevertheless, all these results led to the working hypothesis that the C_4_-associated CA in *Flaveria* evolved via the loss of the sequence coding for the chloroplast transit peptide from the C_3_ CA3 orthologue (Tanz *et al.*, 2009).

Here we present unequivocal evidence that the *ca3* gene of C_4_*F. bidentis* encodes the C_4_-associated CA isoform. We show the *ca3* gene is preferentially expressed in M cells, and the encoded protein localises to the cytosol of M cells. Moreover, our initial experiments on the identification of regulatory sequences controlling *ca3* gene expression show the 2.1 kb region upstream of the translation start of the genes encoding CA3 in C_4_*Flaveria* spp. contains a sequence similar to the MEM1 motif found in the promoter regions of C_4_*Flaveria ppcA* genes. The *ca3* MEM1-like motif directs M cell-specific expression of the *β*-glucuronidase (GUS) reporter gene and, in combination with other elements in the upstream region, confers relatively high levels of reporter gene expression.

## Materials and methods

### *Transformation of* Flaveria bidentis

*Flaveria bidentis* was transformed as described by Chitty *et al.* (1994) using *Agrobacterium tumefaciens* strain AGL1 (Lazo *et al.*, 1991). Integration of the chimerical genes into the *F. bidentis* genome was examined by PCR.

### Mesophyll and bundle-sheath translatomes

*F. bidentis* plants were transformed with constructs that contained either the M-specific *ppcA* promoter of *Flaveria trinervia* (Stockhaus *et al.*, 1997) or the BS-specific promoter of the gene encoding the glycine decarboxylase P subunit (*GLDPA*) from *F. trinervia* (Engelmann *et al.*, 2008) fused to a His(6)-FLAG-tag and the coding sequence of one of the two ribosomal protein RPL18 genes of *F. bidentis* in the binary vector pBI121 (Jefferson *et al.*, 1987).

The ppcA-L-Ft and GLDPA-Ft constructs described previously by Stockhaus *et al.* (1994) and Engelmann *et al.* (2008), respectively, were used as the starting points for the generation of the translatome constructs. Both were digested with *Xma*I and *Sac*I to remove the *uidA* gene from the vector backbone. The His(6)-FLAG tagged *FbRPL18* sequence was generated via PCR and the primers FbRPL18_fw and FbRPL18_rv (Supplementary Table S1 at *JXB* online). His(6)-FLAG-tag and restriction sites were added using PCR and overlapping extended primers (FbRPL18_rv_*Sac*I, Tag1_FbRPL18_fw and Tag2_FbRPL18_fw_*Xma*I; Supplementary Table S1). The final PCR fragment was inserted into pJet1.2/blunt with the CloneJET PCR Cloning Kit (Clontech), and its sequence confirmed. The plasmids were then digested with *Xma*I and *Sac*I, and the inserts introduced into pBI121 containing either the *ppcA* or the *GLDPA* promoter.

Purification of cell-specific polysomes and RNA isolation from mature leaves harvested before the onset of flowering were performed as described previously (Zanetti *et al.*, 2005; Mustroph *et al.*, 2009; Reynoso *et al.*, 2015). The polysome extraction buffer, bead wash buffer, wash buffer and elution buffer were prepared as described by Reynoso *et al.* (2015). RNA isolation was performed by adding 2 volumes of 8 M guanidine-HCl and 3 volumes of 100% ethanol to the eluate, followed by an overnight incubation at −20 °C and 45 min of centrifugation at 15 000 *g* at 4 °C. After washing with 70% ethanol and resuspension in 100 µl H_2_O, a subsequent purification of the RNA with the RNeasy Plant Mini Kit (Qiagen) was performed as described by Mustroph *et al.* (2009).

RNA concentrations were measured with the NanoDrop ND-1000 (NanoDrop Technologies), and 20 ng was reverse transcribed with the QuantiTect Reverse Transcription Kit (Qiagen), following the manufacturer’s protocol. Reverse transcription quantitative PCR (RT-qPCR) was performed with a 7500 Fast Real Time machine (Applied Biosystems), and the KAPA SYBR® FAST qPCR Kit (KAPA Biosystems) using a 100-fold dilution of the cDNA and gene specific primers for CA (CAS_fw and CAS_rv; Supplementary Table S1), PPDK (PPDK_fw and PPDK_rv; Supplementary Table S1), and GLDPA (GLDPA_fw and GLDPA_rv; Supplementary Table S1). The denaturation step was for 3 min at 95 °C, followed by 40 cycles with a two-step setting of 95 °C for 3 s and 60 °C for 30 s. The delta-delta-*C*_t_${\text{(2}}_{}^{-\Delta \Delta C_{\text{t}}})$ method (Livak and Schmittgen, 2001) was used to analyse the relative amount of cDNAs in M-enriched, BS-enriched RNA, and total leaf RNA (from the same isolation as the cell-type-enriched RNAs). The *F. bidentis* actin gene was used as an internal reference gene (Actin_fw and Actin_rv; Supplementary Table S1). Reactions were done in triplicate.

### Flaveria bidentis *CA3 subcellular localisation*

The sequence encoding the ORF (stop codon removed) of *F. bidentis* CA3 was amplified from a pBluescript-CA3 template (Tanz *et al.*, 2009) using the primers MS33-*Xba*I-F and MS34-*Asc*I-R (Supplementary Table S1). The product was digested with *Xba*I and *Asc*I and subcloned into the corresponding sites of the binary vector pMDC83 (Curtis and Grossniklaus, 2003) to produce the plasmid pMDC83-CA3Fbid:GFP.

Transformation and growth of *Agrobacterium tumefaciens* GV3101(pMP90) (Koncz and Schell, 1986) cells, as well as the growth, *Agrobacterium*-infiltration of *Nicotiana benthamiana*, subsequent protoplast preparation and confocal microscopy were carried out as described by Rolland *et al.* (2016). Green fluorescent protein (GFP) and chlorophyll were excited at 488 nm and emission was recorded at 499–535 and 630–735 nm, respectively.

### Flaveria *spp. genome walking*

Genomic DNA was isolated from *F. bidentis* and *Flaveria pringlei* following the method of Marshall *et al.* (1996), and that from *F. trinervia* was isolated according to Gowik *et al.* (2004). Genome walking libraries for *F. bidentis* and *F. pringlei* (Universal Genomewalker), and *F. trinervia* (Universal GenomeWalker 2.0) were constructed according to the manufacturer’s instructions (Clontech).

To obtain the upstream regions of the *F. bidentis* and *F. pringlei ca3* genes, adaptor primers (Clontech), and the *F. bidentis* and *F. pringlei* gene specific primers SAN15 and SAN14 (Supplementary Table S1), respectively, were used in the initial genome walking PCRs according to the manufacturer’s instructions (Clontech). Both primers hybridised to the coding regions of the respective *ca3* genes, between 60 and 85 bp downstream of the translation start sites. Subsequent genome walking assays were done using the Clontech adaptor primers and gene specific primers designed from the 5′-sequences of fragments obtained in previous walking steps. Fragments of 4333 and 2256 bp upstream of the *ca3* translation start codons were isolated for *F. pringlei* and *F. bidentis*, respectively.

An initial 900 bp fragment of the *F. trinervia ca3* gene upstream region was isolated using adaptor primers (Clontech) and the primer EN3-R (Supplementary Table S1), which hybridised in the coding region of the *F. trinervia ca3* gene. A forward primer was then designed, based on the sequence 5′ to the *F. bidentis* MEM1-like element (MS106-F; Supplementary Table S1), and used in combination with EN6-R, which hybridised at the 5′-end of the product of the first walk. This resulted in the amplification of a 1540 bp fragment, which included the sequence encoding the *F. trinervia ca3* MEM1-like motif. To confirm the isolated fragments were contiguous, a PCR using MS112-R and MS113-F primers (Supplementary Table S1) resulted in a 1320 bp fragment that shared a 420 bp overlap with the fragment amplified with MS106 and EN6-R, and extended to 10 bp upstream of the *F. trinervia ca3* translation start.

### Cloning of promoter–reporter gene constructs

A 2114 bp fragment upstream of the translation start site of the *F. bidentis ca3* gene was amplified with primers CA3-1 and CA3-2 (Supplementary Table S1). The primers contained the restriction sites *Sma*I (CA3-1) and *Hin*dIII (CA3-2) that were used to fuse the promoter to the gene encoding GUS in the plant transformation vector pBI121 (construct ca3Fb). For the construct ca3Fb-1.8, which did not contain the MEM1-like motif, a 1872 bp fragment of the *F. bidentis ca3* upstream region was amplified with primers CA3_1 and CA3_3 (Supplementary Table S1), and inserted into pBI121. To fuse the MEM1-like motif to the PR of the *F. trinervia ppcA* promoter (construct ca3Fb-ppcAFtPR), a 74 bp fragment of the *ca3* upstream region containing the MEM1-like motif, from −1943 to −1869, with respect to the *ca3* AUG, was amplified with primers CA3_4 and CA3_5. The primers contained the restriction sites *Xba*I (CA3_4) and *Hin*dIII (CA3_5) that were used to insert the fragment adjacent to the *F. trinervia ppcA* PR in the construct ppcA-S-Ft (in pBI121) described in Stockhaus *et al.* (1994).

### In situ *detection of β-glucuronidase and fluorimetric activity measurements*

Fluorimetric measurements of GUS activity were performed according to Jefferson *et al.* (1987) and Kosugi *et al.* (1990). The statistical significance of the difference between two data sets was analysed using the Mann–Whitney *U* test (Mann and Whitney, 1947). Before the onset of flowering, the fifth leaf of 40- to 50-cm tall T0 *F. bidentis* plants was harvested for the analyses. Histochemical GUS staining and light microscopy were performed as described by Engelmann *et al.* (2008).

### Accession numbers

Sequence information reported in this manuscript can be found in GenBank at the National Center for Biotechnology Information under accession numbers KY239618, KY239617, and KY239619 for the upstream regions of *F. pringlei*, *F. bidentis*, and *F. trinervia*, respectively.

## Results

### Flaveria bidentis *carbonic anhydrase 3 is expressed in the cytosol of mesophyll cells*

**Fig. 1.**
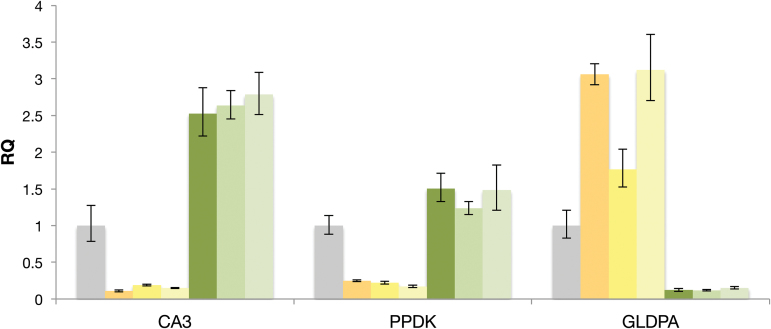
Relative quantification of *Flaveria bidentis* carbonic anhydrase 3 transcripts in leaf cell types. Relative quantification (RQ) of *F. bidentis* transcripts encoding carbonic anhydrase 3 (CA3), pyruvate orthophosphate dikinase (PPDK) and glycine decarboxylase P protein (GLDPA) associated with polysomes from the bundle-sheath cells of three individuals (yellow columns) and mesophyll cells of three individuals (green columns). Transcripts of the reference sample, i.e. polysome-associated RNA from whole leaves, were set to 1 (grey columns). Error bars represent three technical replicates.

Previous work showed *ca3* transcripts are the most abundant CA mRNAs in *F. bidentis* leaves (Tetu *et al.*, 2007); however, the cell type in which the transcripts accumulated was not resolved. *F. bidentis* plants transformed with a construct encoding an epitope tagged ribosomal protein combined with affinity chromatography showed that the mRNA coding for CA3 is highly enriched in polysome complexes from leaf M cells (Fig. 1). The relative enrichment in the three individual plants examined was at least 45% greater than that of transcripts encoding PPDK, which were used as the control for M cell translation complexes. In contrast, the *ca3* transcripts captured in association with leaf BS polysomes from three individual *F. bidentis* plants are depleted to 10% or less, whereas transcripts encoding GLDPA, show up to a three-fold enrichment in epitope-tagged polysomes isolated from BS cells (Fig. 1).

**Fig. 2.**
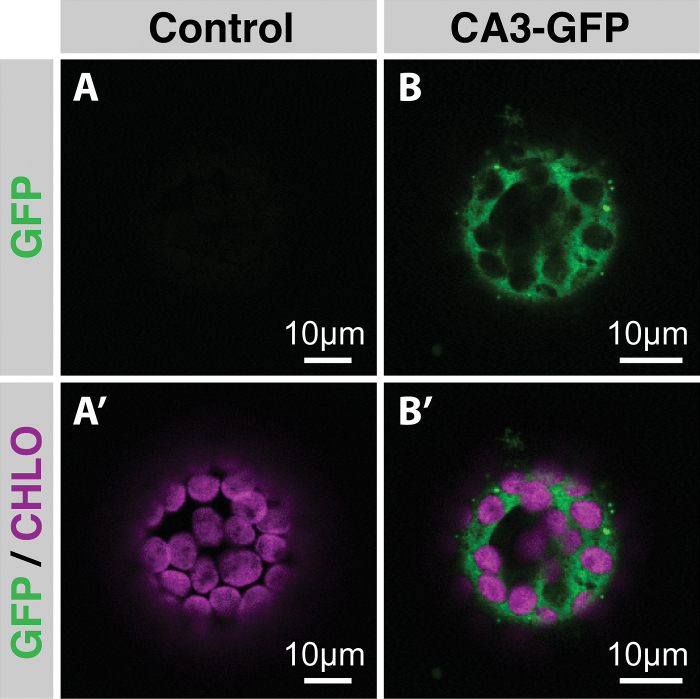
Subcellular localisation of *Flaveria bidentis* carbonic anhydrase 3. (A, A′) Single plane through a protoplast isolated from an *N. benthamiana* leaf that does not express GFP (A), but shows chlorophyll autofluorescence from each chloroplast (A′). (B, B′) Single plane through a protoplast from an *N. benthamiana* leaf expressing CA3–GFP, demonstrating that the GFP signal (B) is cytosolic and does not overlap the chlorophyll autofluorescence emitted from the chloroplasts (B′).

Earlier studies using radiolabelled CA3 proteins from *F. bidentis* and its congener the C_3_*F. pringlei*, and isolated pea chloroplasts demonstrated that unlike *F. pringlei* CA3, the isoform from *F. bidentis* was not recovered in the chloroplast fraction after the import period (Tetu *et al.*, 2007; Tanz, *et al.*, 2009). It was concluded that *F. bidentis* CA3 is a cytosolic M protein; however, it could not be ruled out that it localised to another organelle or a membrane system in M cells. To definitively show its subcellular location, *N. benthamiana* leaves were transformed via infiltration with *Agrobacterium* containing constructs encoding the ORF of *F. bidentis* CA3 fused to that of GFP. Protoplasts isolated 2 days post-infiltration from untransformed *N. benthamiana* leaves showed only chlorophyll autofluorescence (Fig. 2A, A′). In contrast, protoplasts expressing the CA3–GFP fusion protein showed a GFP signal that did not co-localise with the chlorophyll autofluorescence of the chloroplasts, but instead clearly surrounded each of the chloroplasts, indicating a cytosolic location (Fig. 2B, B′).

### *The upstream regions of* ca3 *from C_4_* Flaveria *species contain a MEM1-like element*

**Fig. 3.**
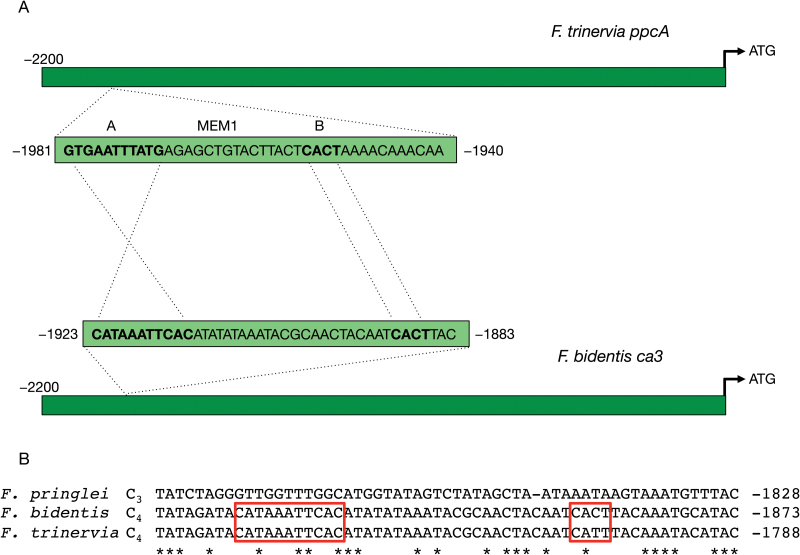
Structures and sequences of mesophyll expression module 1 (MEM1) and MEM1-like elements. (A) The mesophyll expression module 1 (MEM1) and MEM1-like elements of C_4_*Flaveria ppcA* and *ca3* genes, respectively, are located within the first 2 kb upstream of the translation start sites of the proteins. The 41 bp elements consist of A and B segments, with the sequence of the *ca3* A segment inverted relative to that of *ppcA*. (B) The B segments of the *F. bidentis ca3* MEM1-like element, like the C_4_*Flaveria ppcA* MEM1, encodes a CACT tetranucleotide; however, the corresponding region of the MEM1-like B segment from *F. trinervia ca3*, is a CATT tetranucleotide. Little sequence homology is seen in the comparable upstream region of the *ca3* gene from the C_3_ species *F. pringlei*. (This figure is available in colour at *JXB* online.)

Genome walking was used to isolate the 5′-region of the *ca3* genes from *F. bidentis* and *F. pringlei*, with the aim of identifying *cis*-acting motifs responsible for the differences in expression levels and patterns of the C_3_ and C_4_ orthologues. Sequence determination of the ~2.1 kb region upstream of the translation start site of the *F. bidentis ca3* gene revealed a 41 bp fragment with segments showing high sequence identity to the MEM1 element responsible for M-specific expression of the *ppcA* gene, which codes for the C_4_-associated PEPC (Fig. 3; Gowik *et al.*, 2004). The *F. bidentis ca3* MEM1-like sequence consists of A and B segments homologous to those of the *ppcA* MEM1; however, the sequence of the *ca3* A segment is inverted relative to that of the *ppcA* element (Fig. 3). The *F. bidentis ca3* MEM1-like B segment shows little sequence identity to the *ppcA* Mem1 B segment, except for the CACT tetranucleotide (Fig. 3; Gowik *et al.*, 2004). A MEM1-like element is also found in the comparable upstream region of the *ca3* gene from another C_4_*Flaveria* species, *F. trineriva*; however, while the sequences of the two *ca3* MEM1-like A segments are identical, the tetranucleotide in the B segment in *F. trinervia* is CATT (Fig. 3).

In contrast to the two C_4_*Flaveria* species, the 2 kb upstream of the translation start site of *ca3* from the C_3_ species, *F. pringlei*, does not contain a sequence with homology to either the MEM1 A or B segments (Fig. 3 and Supplementary Fig. S1). As a consequence, the sequence of the *F. pringlei ca3* upstream region was extended a further 2 kb upstream; however, still no homology was found with C_4_ MEM1 or MEM1-like elements (data not shown). In fact, the *F. pringlei ca3* upstream region shares only limited blocks of sequence homology with the corresponding regions of the two C_4_ species (Supplementary Fig. S1).

### *The MEM1-like element of the* Flaveria bidentis *carbonic anhydrase 3 gene directs expression in mesophyll cells*

**Fig. 4.**
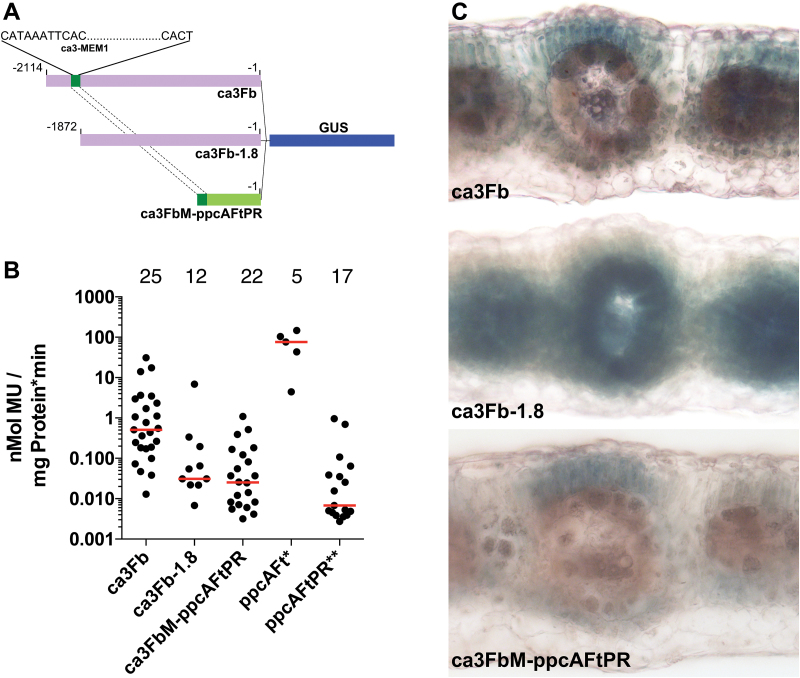
Analysis of *Flaveria bidentis* promoter–reporter gene constructs. (A) Structures of the *ca3*–*β*-glucuronidase (GUS) chimerical genes: ca3Fb represents the 2114 bp region upstream of the *ca3* translation start site, and includes the MEM1-like element; ca3Fb1.8 denotes the 1872 bp upstream of the *ca3* translation start site; ca3FbM-ppcAFtPR designates the *F. bidentis ca3* MEM1-like element fused to the proximal promoter region (PR) of the *F. trinervia ppcA* gene. (B) GUS activities in leaves of transgenic *F. bidentis* plants. Data for constructs ppcAFt (*) and ppcAFtPR (**) were taken from Gowik *et al.* (2004). Median values are indicated as red bars. Numbers above the values represent the number of individual plants assayed. Mu, 4-methylumbelliferone. (C) Histochemical localisation of GUS activity in leaf sections of transgenic *F. bidentis* plants transformed with the ca3Fb, ca3-1.8 and ca3Fb-ppcAFtPR constructs. Incubation times for GUS staining were 8 h (ca3Fb), 24 h (ca3Fb-1.8.) and 26 h (ca3FbM-ppcAFtPR).

To test whether the MEM1-like element of the *F. bidentis ca3* upstream region, like the *ppcA* MEM1, is capable of conferring M cell-specific expression, *F. bidentis* wild type plants were transformed with constructs containing parts of the ~2.1 kb upstream region from the *F. bidentis ca3* gene fused with the GUS reporter gene (Fig. 4A). When the entire ~2.1 kb fragment, which contained the MEM1-like element (ca3Fb), was used in the reporter construct, GUS activity in the leaves of transformants was approximately 16 times greater than when the upstream fragment without the MEM-1-like sequence (ca3Fb-1.8) was fused to GUS (Fig. 4B). This difference is significant as judged by the Mann–Whitney *U* test (*P* = 0.0007). By comparison, GUS activity in leaves of *F. bidentis* plants that were transformed with the *F. trinervia ppcA* promoter containing the MEM1 sequence (ppcAFt; Stockhaus *et al.*, 1997) was more than two orders of magnitude and significantly (*P* < 0.0001) greater than the activity found with ca3Fb (Fig. 4B). Approximately 3.5 times more GUS activity was found in the leaves of transformants when the *F. bidentis ca3* MEM1-like sequence was fused to the PR of the *ppcA* gene (ca3FbM-ppcAFtPR; Fig. 4B) relative to the PR alone (ppcAFtPR; Fig. 4B), although this difference was not significant (*P* = 0.2289). However, the level of activity was of the same magnitude as the relatively low activity found for the ca3Fb-1.8 construct (Fig. 4B).

Histochemical staining of GUS activity in the leaves of ca3Fb transformants was detected only in M cells (Fig. 4C). In contrast, no cell-specific GUS staining was seen in the leaves of plants transformed with ca3Fb-1.8; instead staining was detected in M and BS cells, as well as in the vascular tissue (Fig. 4C). Although the fluorometric assays indicated relatively low GUS activity in the leaves of plants transformed with the ca3 MEM1-like sequence fused to the PR of the *ppcA* gene (ca3FbM-ppcAFtPR), histochemical localisation of GUS activity in the leaves was detected only in the M (Fig. 4C). In contrast, transformants containing constructs with only the *ppcA* PR also demonstrated GUS activity in the BS and vasculature (Akyildiz *et al.*, 2007).

We conclude from these initial promoter analyses that the MEM1-like motif of the *F. bidentis ca3* gene acts as an enhancer of gene expression preferentially in the M cells of *F. bidentis* as an increase in GUS activity was observed when the motif was present in the transformation construct relative to its absence (Fig. 4B). However, the element also appears to inhibit GUS activity in the BS and vascular tissues when it is present (Fig. 4C). While the levels of GUS activity in plants transformed with ca3FbM-ppcAFtPR were similar to those of the *ca3* upstream region without the MEM1-like element, histochemical staining showed the presence of the element conferred M-specific GUS expression (Fig. 4B, C). Taken together, these results indicate that the MEM1-like element of the *F*. *bidentis ca3* gene is a *cis*-acting element that directs M cell-specific expression.

## Discussion

The C_4_ photosynthetic pathway has evolved independently from C_3_ ancestors in at least 65 different angiosperm lineages (Sage *et al.*, 2012; Sage, 2016). This suggests, in terms of molecular genetics, that it is a relatively easy conversion (Gowik *et al.*, 2004). In a current model of C_4_ evolution, the steps that include the strict compartmentation of enzymes between M and BS and the optimisation of the pathway, with the accompanying evolution of the regulatory elements controlling these processes, are considered to occur during the later stages of the transition (Sage *et al.*, 2012). Increasing evidence indicates that distinct mechanisms control the expression patterns and levels of genes encoding C_4_ isoforms. Modifications to sequences in ancestral C_3_ promoter and untranslated regions (UTRs), as well as introns, control levels of gene expression, while different motifs in promoters, exons and UTRs direct cell-specific patterns of expression (Ludwig, 2013; Heimann *et al.*, 2013; Williams *et al.*, 2016).

### Flaveria bidentis ca3 *encodes the carbonic anhydrase associated with the C_4_ pathway*

In this study, we have focused on the absolute identification of the gene encoding the CA isoform that catalyses the first step in the C_4_ pathway in *Flaveria* and the elements controlling its expression. Previous work on CA in the C_4_ species *F. bidentis* strongly supported a C_4_-associated role for the CA3 isoform (von Caemmerer *et al.*, 2004; Tetu *et al.*, 2007; Tanz *et al.*, 2009). In the present study, we have extended these results and have shown unequivocally that the *F. bidentis ca3* gene is expressed preferentially in leaf M cells and it encodes a cytosolic form of CA.

Previous results of RT-qPCR assays indicated that mRNAs encoding CA3 are at least 50 times more abundant than those coding for CA1 or CA2 in *F. bidentis* leaves (Tetu *et al.*, 2007), and more than 10 times greater than any of the CA transcripts from the C_3_*F. pringlei* on a leaf total RNA basis (Ludwig, 2011). While these high expression levels argued that the *ca3* gene most likely encoded the CA isoform associated with the C_4_ pathway in *Flaveria*, they did not show whether the accumulation of *ca3* transcripts was specifically in the M cells, as anticipated for a C_4_ species (Gutierrez *et al.*, 1974; Ku and Edwards, 1975; Burnell and Hatch 1988). Results of translatome experiments in the present study conclusively demonstrated that *ca3* transcripts are highly enriched in the polysome fraction of *F. bidentis* M cells, being about 15 times greater than in the translation complexes of BS cells, and about twice the abundance of the mRNA encoding the C_4_-associated PPDK that functions in M cells (Fig. 1).

Having established the *F. bidentis ca3* gene is preferentially expressed in M cells, we then set out to definitively show that its cognate protein has a cytosolic location, which is essential for the provision of HCO_3_^−^ to PEPC and C_4_ pathway function. Tetu *et al.* (2007) showed, with import studies using isolated pea chloroplasts, that the *F. bidentis* CA3 was not imported into the isolated organelles, unlike the CA3 homologue from the C_3_ species *F. pringlei* (Tanz *et al.*, 2009). It was concluded that *F. bidentis* CA3 is a cytosolic protein; however, as cytosolic fractions could not be isolated in these import studies, there was no direct evidence for this conclusion. Here we demonstrated that when the coding region of *F. bidentis* CA3 is fused to that of GFP and used to transform *N. benthamiana* leaves, GFP fluorescence is unequivocally cytosolic in protoplasts from these leaves (Fig. 2). GFP signal clearly surrounds the chlorophyll autofluorescence emitted from the chloroplasts, with no overlap in these fluorescence signals.

The translatome and the localisation results of the present study substantiate the proposal that the loss of the sequence encoding the chloroplast transit peptide of the ancestral C_3_*Flaveria* CA3 protein enabled the evolution of the C_4_ form of the enzyme, by trapping it in the M cytosol (Tanz *et al.*, 2009). In addition, they corroborate the finding that reduction of CA3 in *F. bidentis* through antisense technology leads to a significant impairment of the CCM in this C_4_ species (von Caemmerer *et al.*, 2004).

### *The MEM1-like element of the* F. bidentis ca3 *gene, like the C_4_*Flaveria ppcA* MEM1, directs mesophyll cell-specific expression*

Like the MEM1 element of the *F. trinervia* and *F. bidentis ppcA* genes (Gowik *et al.*, 2004; Akyildiz *et al.*, 2007), the MEM1-like motif of C_4_*Flaveria ca3* genes is located about 2 kb upstream of the translation start site. In contrast, while homologous sequences can be identified 2–2.5 kb upstream of the translation start sites of C_3_ and C_4_*Flaveria ppcA* orthologues (Gowik *et al.*, 2004; Akyildiz *et al.*, 2007), the *ca3* upstream region from the C_3_ species *F. pringlei* shows no sequence similarity to the C_4_*F. bidentis* and *F. trinervia* 5′-regions in the vicinity of the MEM1-like motif (Supplementary Fig. S1). As the upstream regions of the *ppcA* genes from *Flaveria* congeners show insertions and deletions relative to one another, we determined the sequence of a further 2 kb upstream of the *F. pringlei ca3* gene; however, we found no evidence of a MEM1-like sequence in this part of the genome.

The structures of the *ppcA* MEM1 and *ca3* MEM1-like motifs are highly similar, with recognisable A and B segments in the MEM1-like elements (Fig. 3); however, the sequence of the MEM1-like A segments is the reverse complement of the sequence encoding the A segments of the C_4_*ppcA* MEM1. The MEM1 B segment of C_4_, C_4_-like and C_3_–C_4_*Flaveria ppcA* genes has an invariant CACT tetranucleotide, which is not seen in the orthologues of C_3_ congeners (Gowik *et al.*, 2004). A CACT sequence is found 23 bp downstream of the MEM1-like A segment in the *F. bidentis ca3* upstream region while a CATT tetranucleotide is found in the corresponding position of the C_4_*F. trinervia* MEM1-like motif. The distal promoter region encoding the *ppcA* MEM1, in combination with the PR of the *ppcA* promoter, was found to direct M-specific GUS expression in both sequence orientations, characteristic of a transcriptional enhancer (Gowik *et al.*, 2004). This activity supports the evidence presented here that shows the MEM1-like motif also acts as an enhancer, conferring a higher level of GUS expression when present with the ca3Fb-1.8 region or when fused to the *F. trinervia ppcA* PR (Fig. 4).

Histochemical localisation of GUS activity showed preferential staining of the M cells in leaves of transformed *F. bidentis* plants when the ~2.1 kb upstream region of the *F. bidentis ca3* gene was included in the transformation construct (ca3Fb; Fig. 4C). This M-specific staining pattern was also found when only the MEM1-like region was used in combination with the PR of the *F. trinervia ppcA* gene (ca3FbM-ppcAFtPR; Fig. 4C). In contrast, no cell-specificity in GUS staining was seen when the *F. bidentis ca3* upstream region without the MEM1-like motif (ca3Fb-1.8; Fig. 4C), or just the PR of the *F. trinervia ppcA* gene (Akyildiz *et al.*, 2007) was used to transform *F. bidentis* plants.

From these experiments we can conclude that the *ca3* MEM1-like motif resembles the C_4_*Flaveria ppcA* MEM1 element not only in its structure but also in its function as it preferentially directs M expression of the GUS reporter gene and acts as an enhancer of expression in the M. Moreover, the MEM1-like motif also functions to repress transcriptional activity in the BS cells, as well as in other leaf cell types.

The GUS activity levels in the leaves of ca3Fb transformants are at least two orders of magnitude less than those of *F. bidentis* transformants containing the promoter region of the *F. trinervia ppcA* gene (ppcAFt; Fig. 4B). This difference may be attributed to additional promoter elements not in the *ca3* ~2.1 kb 5′-region. Alternatively it may reflect a true difference in the strengths of the two promoters that could imply additional post-transcriptional regulation of transcript levels. An antisense construct targeted against *F. bidentis ca3* transcripts showed that although CA activity in wild type *F. bidentis* plants does not limit photosynthesis, relatively high activity levels are required for the CCM to function properly in this C_4_ dicot (von Caemmerer *et al.*, 2004). Although care needs to be taken in extending transcriptional activity with either protein abundance or activity (Vélez-Bermúdez and Schmidt, 2014), it is likely that the MEM1-like element and its associated transcription factors are not the only mechanism ensuring sufficient CA activity is present to support the provision of HCO_3_^−^ for PEPC. As our current understanding of C_4_ gene expression expands, we need to consider control at the transcriptional level involving epigenetic marks, and post-transcriptional mechanisms at the level of both the transcript and the protein.

### *Evolution of C_4_ related* cis-*regulatory elements and gene regulation*

It is well known that the expression patterns of most of the genes encoding proteins involved in C_4_ photosynthesis changed during C_4_ evolution as overall expression was enhanced and many of these genes acquired either M- or BS-specific expression. However, the modifications in gene structure responsible for these changes in expression have been identified at the molecular level for only a few of these genes (Rosche *et al.*, 1998; Nomura *et al.*, 2000; Gowik *et al.*, 2004; Brown *et al.*, 2011; Heimann *et al.*, 2013; Williams *et al.*, 2016).

Interestingly, recent studies have shown that in different C_4_ lineages, several genes encoding C_4_-associated proteins appear to be controlled, at least partially, by common mechanisms and *cis*-regulatory motifs. Common histone modifications that control the expression of genes encoding multiple C_4_-associated proteins have been identified in different grass C_4_ lineages. In maize, *Sorghum bicolor* (sorghum) and *Setaria italica*, light-regulated acetylation of histone H3 at K9 was found to be a shared histone mark in the promoter regions of genes encoding the C_4_-associated PEPC and NADP-ME, and in maize this modification was also observed in the promoter regions of genes encoding the C_4_ forms of CA, PCK and PPDK (Heimann *et al.*, 2013). Cell-specific regulation of trimethylation of K4 on histone H3 was a common modification in these lineages for a number of genes encoding C_4_-associated enzymes, including maize CA (Heimann *et al.*, 2013).

In the coding regions of NAD-malic enzyme and NADP-ME subunit genes from different C_4_ lineages, homologous sequences have been isolated that confer BS-specific reporter gene expression (Brown *et al.*, 2011). More recently, Williams *et al.* (2016) described a nine-nucleotide motif that is found in the 3′- and 5′-UTRs of *GgCA4*, the C_4_-associated CA of *Gynandropsis gynandra*. This sequence, designated MEM2, in combination with an element in the *G. gynandra* PR is sufficient to direct high levels of the GUS reporter gene preferentially in M cells. MEM2 motifs are also found in the 3′- and 5′-UTRs of the gene encoding the C_4_-associated PPDK in *G. gynandra*, as well as in the 3′-end of the gene coding for *GgCA2* (Williams *et al.*, 2016).

Here we demonstrated that the MEM1-like element of the *F. bidentis ca3* gene shares the regulatory function of directing M cell-specific expression with the C_4_*Flaveria ppcA* gene MEM1 motif. This implies that these motifs were already established within the promoter sequences when these genes were recruited to the C_4_ pathway, bringing both genes under the control of a common *trans*-regulatory network that might have also existed in the last non-C_4_*Flaveria* ancestors.

In case of the *Flaveria ppcA* promoter it appears that MEM1 evolved step by step from an ancestral C_3_ motif via point mutations as well as insertions and deletions of short DNA stretches (Gowik *et al.*, 2004; Akyildiz *et al.*, 2007). Sequences very similar to that of MEM1 and the regions surrounding it, but not functional in M-specific gene expression, are found in the *ppcA* promoters of C_3_*Flaveria* species (Gowik *et al.*, 2004; Akyildiz *et al.*, 2007), indicating that the C_4_ MEM1 evolved from a C_3_ predecessor (Gowik *et al.*, 2004). The *ppcA* genes of *Flaveria* are thought to have originated from the duplication of an ancestral *ppcB*-like gene long before the emergence of C_4_ photosynthesis in this genus (Svensson *et al.*, 2003). Importantly, sequences with obvious similarity to MEM1 have been identified in the promoter regions of *ppcB* genes from C_3_ and C_4_*Flaveria* species (Akyildiz *et al.*, 2007). This implies that a MEM1-related sequence in *Flaveria ppc* promoters was an ancestral motif that was recruited for function in C_4_ photosynthesis after some modification and optimisation.

The situation is quite different for the *Flaveria ca3* genes. The sequences surrounding the MEM1-like motifs are highly conserved in the two C_4_ species, but cannot be identified in the 4 kb *ca3* upstream region from the C_3_ species *F. pringlei*. This implies that the MEM1-like motif was not part of the ancestral *Flaveria ca* genes, but instead was acquired before or during C_4_ evolution in the genus by recombination. Alternatively, the motif may have been lost from the predecessor of C_3_*ca3* genes after the relatively recent divergence of C_3_ and C_4_*Flaveria* species (Lyu *et al.*, 2015).

Ascertaining the scenario by which the MEM1-like element was acquired for C_4_*Flaveria ca3* gene expression will be possible once genome sequences of C_3_ and C_4_*Flaveria* congeners are available. The distribution of MEM1-like motifs in the genomes could be examined, and putative recombination events could be reconstructed. Importantly, the possible spreading of *cis*-regulatory element precursors within the genome with subsequent modifications and recruitment to C_4_-related gene regulation could be investigated. These types of comparative studies will provide insights and a potential mechanism into how similar changes in the expression patterns of several genes during C_4_ evolution has been realised in multiple C_4_ lineages.

## Conclusion

In the cytosol of C_4_ M cells, the enzymes CA and PEPC catalyse the first two reactions of the C_4_ photosynthetic pathway, regardless of C_4_ subtype (Hatch 1987; Hatch and Burnell, 1990), or whether a plant uses Kranz anatomy or a single-celled C_4_ system (Offermann *et al.*, 2011). As the activity of PEPC is dependent on HCO_3_^−^, the product of CA catalysis, it is conceivable that during the evolution of C_4_ photosynthesis in 65 (or more) angiosperm lineages, a similar regulatory mechanism was adopted to ensure the coordinated expression of the cognate genes. As shown here, this appears to be the case in *Flaveria*.

The present study has built on previous work (von Caemmerer *et al.*, 2004; Tetu *et al.*, 2007, Tanz *et al.*, 2009) to conclusively show the *ca3* gene from *F. bidentis* encodes the CA associated with the C_4_ pathway. Our results indicate that the *ca3* MEM1-like element, like the *ppcA* MEM1, is sufficient and required for M-specific promoter activity. They also suggest that distinct mechanisms control this cell-type expression pattern and the activity of the *ca3* gene promoter. In all likelihood additional transcriptional as well as post-transcriptional control mechanisms are required to provide sufficient CA activity to support the *F. bidentis* C_4_ CCM.

## Supplementary data

Supplementary data are available at *JXB* online.

Fig. S1. Multiple sequence alignment of C_3_ and C_4_*Flaveria* carbonic anhydrase 3 upstream regions.

Table S1. Primers used in this study.

## Supplementary Material

Supplementary_Figure_S1_Table_S1

## Abbreviations:

BS: bundle-sheath
CA: carbonic anhydrase
CCM: CO_2_ concentrating mechanism
GFP: green fluorescent protein
GLDPA: glycine decarboxylase P protein
GUS: *β*-glucuronidase
M: mesophyll
MEM: mesophyll expression module
NADP-ME: NADP-malic enzyme
PCK: phospho*enol*pyruvate carboxykinase
PEPC: phospho*enol*pyruvate carboxylase
PPDK: pyruvate orthophosphate dikinase
PR: proximal promoter region.
