# Supplementary material for: A MEM1-like motif directs mesophyll cell-specific expression of the gene encoding the C4 carbonic anhydrase in Flaveria
Source: J Exp Bot. 2016 Dec 31;68(2):311–20. doi: 10.1093/jxb/erw475 (PMC5853542; doi:10.1093/jxb/erw475)
Supplement: Supplementary_Figure_S1_Table_S1 [file erw475_suppl_supplementary_figure_s1_table_s1.pdf]

Supplementary Table S1. Primers used in this study.

**Primer Name**	**Sequence 5' to 3'**
FbRPL18_fw	ATGGGTATCGATCTCGTTGC
FbRPL18__rv	TTAGTTTCTAAATCCCCTGC
FbRPL18_rv_*Sac*I	TATGAGCTCTTAGTTTCTAAATCCCCTGC
Tag1_FbRPL18_fw	ATGACGACGATAAGGGTGGTGGTGGTGGTGGTATGGGTATCGATCTCGTTGC
Tag2_FbRPL18_fw_*XmaI*	TATCCCGGGATGCATCATCACCATCACCACGGTGGTGGTGATTACAAGGATGACGACGATAAGGGTGGT
Actin_fw	AATGGAAGCTGCTGGTATTCA
Actin_rv	CAACCACCTTGATCTTCATGC
PPDK_fw	CAAGTTCGTGCGATCTTTCA
PPDK_rv	AGCAGCTCGAGGAATCTCAA
GLDPA_fw	TGGCTGATAAGTGGACCAAA
GLDPA_rv	CCAGAATTTAGCAGCACGAA
CAS_fw	TGAGTGTAATATCTTTTGCAGAGACG
CAS_rv	TGAAGTTGGGCGGTGATTT
CA3_1	GGGAAGCTTACATGCTAAACACATGCATAA
MS33-*Xba*I-F	TCTAGACATGGGAAGTAAATCATATGATGA
MS34-*Asc*I-R	GGCGCGCCATGCCCGGGTAGTAGGTGAA
MS106-F	CACATACACATTGTTTACACCGATACA
SAN14	TCACTTAAAAGCTTCTTAAGTGCAGC
SAN15	TGCTAAGCCTTGCAAAGCAAGTGG
MS112-R	TGCAAACTCACAACTTTTAACTT
MS113-F	ACAAACTAGTTGCTAACTGTGGG
EN3-R	GGGCGGTGATTTGGTCGATTTTTGCAG
EN6-R	GTTGACATATACCATCAGGTTCACCAATCT
CA3_2	ATACCCGGGGTCTCGTCTCTGCAAAAGAT
CA3_3	GGGAAGCTTTGTACACATAAACACATTAA
CA3_4	GGGTCTAGAACATGTATGCATTTGTAAGT
CA3_5	GGGAAGCTTTATGCACGCATGTATAGATA

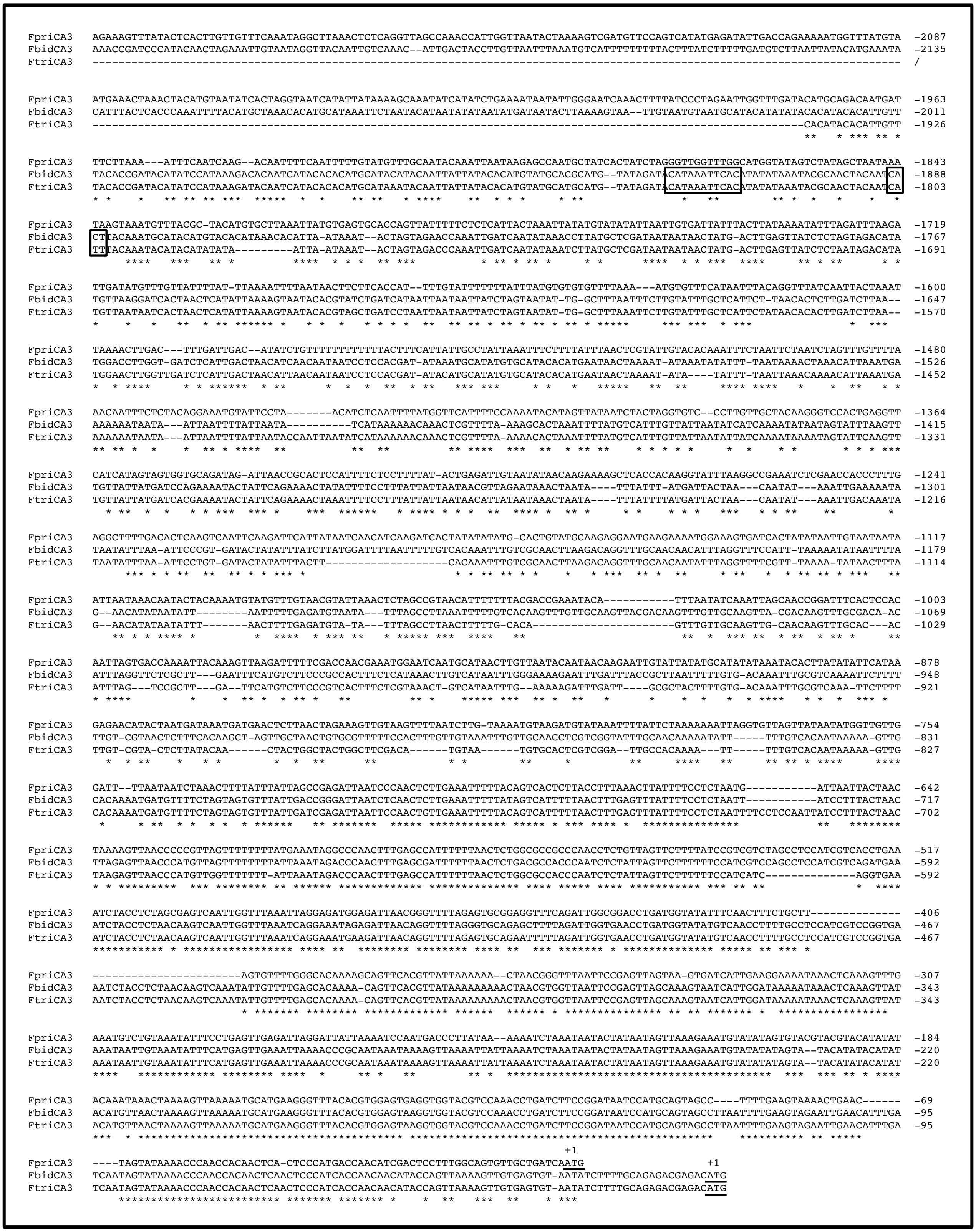

Figure S1. Multiple sequence alignment of C_3 and C_4 *Flaveria* carbonic anhydrase 3 upstream regions. The nucleotide sequences of the *Flaveria pringlei* (C_3), *F. bidentis* (C_4) and *F. trinervia* (C_4) carbonic anhydrase genes were aligned using ClustalW (Thompson et al., 1994). Translation start sites are underlined. The A and B segments of the MEM1-like motifs are boxed. Asterisks indicate nucleotide identities, dashes represent gaps inserted to maximise the alignment.

References Supplementary Material

Thompson JD, Higgins DG, Gibson TJ. 1994. CLUSTALW: improving the sensitivity of progressive multiple sequence alignment through sequence weighting, position-specific gap penalties and weight matrix choice. Nucleic Acids Research **22**: 4673–4680.
